# Changes in patient activation following cardiac rehabilitation using the Active^+^me digital healthcare platform during the COVID-19 pandemic: a cohort evaluation

**DOI:** 10.1186/s12913-021-07363-7

**Published:** 2021-12-24

**Authors:** Gabbi Frith, Kathryn Carver, Sarah Curry, Alan Darby, Anna Sydes, Stephen Symonds, Katrina Wilson, Gordon McGregor, Kevin Auton, Simon Nichols

**Affiliations:** 1 Advanced Wellbeing Research Centre, Sheffield Hallam University, Sheffield, S9 3TY UK; 2 Sport and Physical Activity Research Centre, Sheffield Hallam University, Sheffield, S10 2BP UK; 3 Cardiology Services, Cambridge University Hospitals NHS Foundation Trust, Addenbrooke’s Hospital, Cambridge, CB2 0QQ UK; 4 Eastern Academic Health Science Network, Magog Court, Cambridge, CB22 3AD UK; 5 Cardiac Rehabilitation Service, Cambridge University Hospitals NHS Foundation Trust, Addenbrooke’s Hospital, Cambridge, CB2 0QQ UK; 6 And The Beat Goes On - Phase IV Cardiac Rehabilitation Programme, Cambridge, CB2 9BE UK; 7 Department of Cardiopulmonary Rehabilitation, Centre for Exercise & Health, University Hospitals Coventry & Warwickshire NHS Trust, Coventry, CV2 2DX UK; 8 Warwick Clinical Trials Unit, Warwick Medical School, University of Warwick, Coventry, CV4 7AL UK; 9 Centre for Sport, Exercise & Life Sciences, Coventry University, Coventry, CV1 5FB UK; 10 Aseptika Limited (Activ8rlives), St Ives Business Park, St Ives, Cambridgeshire PE27 4AA UK

**Keywords:** Cardiac rehabilitation, Patient activation, Self-efficacy, Tele-health, COVID-19

## Abstract

**Background:**

Restrictions on face-to-face contact, due to COVID-19, led to a rapid adoption of technology to remotely deliver cardiac rehabilitation (CR). Some technologies, including Active^+^me, were used without knowing their benefits. We assessed changes in patient activation measure (PAM) in patients participating in routine CR, using Active^+^me. We also investigated changes in PAM among low, moderate, and high risk patients, changes in cardiovascular risk factors, and explored patient and healthcare professional experiences of using Active^+^me.

**Methods:**

Patients received standard CR education and an exercise prescription. Active^+^me was used to monitor patient health, progress towards goals, and provide additional lifestyle support. Patients accessed Active^+^me through a smart-device application which synchronised to telemetry enabled scales, blood pressure monitors, pulse oximeter, and activity trackers. Changes in PAM score following CR were calculated. Sub-group analysis was conducted on patients at high, moderate, and low risk of exercise induced cardiovascular events. Qualitative interviews explored the acceptability of Active^+^me.

**Results:**

Forty-six patients were recruited (Age: 60.4 ± 10.9 years; BMI: 27.9 ± 5.0 kg^.^m^2^; 78.3% male). PAM scores increased from 65.5 (range: 51.0 to 100.0) to 70.2 (range: 40.7 to 100.0; *P* = 0.039). PAM scores of high risk patients increased from 61.9 (range: 53.0 to 91.0) to 75.0 (range: 58.1 to 100.0; *P* = 0.044). The PAM scores of moderate and low risk patients did not change. Resting systolic blood pressure decreased from 125 mmHg (95% CI: 120 to 130 mmHg) to 119 mmHg (95% CI: 115 to 122 mmHg; *P* = 0.023) and waist circumference measurements decreased from 92.8 cm (95% CI: 82.6 to 102.9 cm) to 85.3 cm (95% CI 79.1 to 96.2 cm; *P* = 0.026). Self-reported physical activity levels increased from 1557.5 MET-minutes (range: 245.0 to 5355.0 MET-minutes) to 3363.2 MET-minutes (range: 105.0 to 12,360.0 MET-minutes; *P* < 0.001). Active^+^me was acceptable to patients and healthcare professionals.

**Conclusion:**

Participation in standard CR, with Active^+^me, is associated with increased patient skill, knowledge, and confidence to manage their condition. Active^+^me may be an appropriate platform to support CR delivery when patients cannot be seen face-to-face.

**Trial registration:**

As this was not a clinical trial, the study was not registered in a trial registry.

**Supplementary Information:**

The online version contains supplementary material available at 10.1186/s12913-021-07363-7.

## Introduction

In 2020, a highly contagious virus, known as COVID-19 [1], resulted in international governments restricting face-to-face contact [2]. This led to the suspension of ‘non-essential’ healthcare services, including half of cardiac rehabilitation (CR) services in the United Kingdom [3]. However, CR programmes that were not suspended adapted rapidly. Three-quarters (77.6%) of programmes introduced digital platforms to deliver CR for the first time between 11th March 2020 [3], when COVID-19 was declared a pandemic by the World Health Organisation [4], and 20th June 2020 [3]. However, the benefit of some digital platforms on patient outcomes are unknown [3]. Furthermore, evidence suggested that some patient groups, including those at high risk of exercise-induced cardiovascular events [5], were not being offered exercise-based CR using technology [3]. There is as a need to identify which platforms adopted during the COVID-19 pandemic are effective and safe, particularly for high risk patients.

In March 2020, Addenbrooke’s Hospital (Cambridge University Hospitals NHS Foundation Trust, UK) introduced a customisable telemetry system called Active^+^me (Aseptika Ltd, St Ives, UK). Active^+^me was accessed by patients through a tablet or smart phone. Healthcare professionals controlled the content that was available to patients, and communicated with patients, through a PC terminal. Active^+^me was used to monitor and provide additional support to patients participating in a routine, eight-week, comprehensive CR programme [6] that had transitioned to remote service delivery due to the COVID-19 pandemic. Active^+^me was designed to increase patient activation, a term used to describe the level of knowledge, skill, and confidence a patient has to self-management their condition [7]. Patient activation can be measured using the patient activation measure (PAM) questionnaire [8].

Patient activation measure scores are associated with health outcomes. Patients with chronic heart failure (CHF) who are admitted to hospital are 10% more likely to be discharged home, rather than to a ‘skilled nursing facility’, with each 1-point increase in PAM score (odds ratio [OR] 1.08; 95% CI: 1.03 to 1.14, *P* < 0.001) [9]. Conversely, 30 day mortality rates increase by 10% with each 1-point decrease in PAM score, in patients with CHF (hazard ratio 1.09, 95% CI 1.03 to 1.16, *P* = 0.006) [9]. Similarly, patients with coronary heart disease (CHD) with the lowest levels of activation (level 1) are more likely to experience clinically significant declines in mental health (OR 1.95; 95% CI: 1.05 to 3.62), and disease specific health-related quality of life (OR 2.18; 95% CI: 1.17 to 4.05; *P* < 0.05), 1 month after hospital discharge [10]. Thus, increasing patient activation may reduce healthcare costs, improve patient health-related quality of life, and reduce the risk of death, in the short-term.

The primary aim of this cohort study was to determine whether participating in CR, with Active^+^me, led to an increase in PAM score. To help overcome the hesitance of services providing remote CR to high risk patients [3], we also explored changes in PAM scores in patients categorised at high, moderate, and low risk of exercise-induced cardiovascular events [5]. Finally, we investigated patient and healthcare professionals’ experiences of using Active^+^me.

## Methods

### Participants & consent

Ethical approval for the evaluation was obtained from Sheffield Hallam University Ethics Committee (ER26525336). The evaluation was also approved by Addenbrooke’s Hospital (ID 3224; Ref no. PRN9224). Study procedures conform to the 1975 Declaration of Helsinki and is subsequent revisions. Informed consent was obtained by a member of the patient’s immediate healthcare team prior to enrolling patients on to the service evaluation. Consent was recorded in the patient healthcare record. Written informed consent was also obtained for interviews conducted with patients and healthcare professionals. Reporting of findings adhere to STROBE guidelines (Appendix 1) [11].

As this was a service evaluation, no formal sampling strategy was used. Patients referred for routine, 8 week, Phase III CR were sequentially invited to participate in CR with Active^+^me between the 6th April 2020 and 27th July 2020. Patients who were > 18 years of age with a recent diagnosis of atherosclerosis, angina, myocardial infarction (MI), CHF, or had undergone coronary artery bypass graft (CABG) surgery, elective percutaneous coronary intervention (PCI), or valve surgery, were eligible for recruitment. Because this was a routine healthcare service, the only exclusion criterion was an absolute contraindication to exercise training [5]. Patients underwent an initial, remote, holistic assessment by a CR healthcare professional approximately 1 week before commencing CR. Assessments were used to assess lifestyle and medical risk factors [6], and formulate a treatment plan including exercise training. Healthcare professionals scored patients as either high, moderate, or low risk of exercise-induced cardiovascular events using established criteria [5]. A follow-up assessment was conducted approximately 1 week after completing CR. Patients were free to decline Active^+^me. Patients who declined Active^+^me received exercise and lifestyle advice over the telephone a written exercise programme was also provided at the discretion of the healthcare professional, when appropriate.

### Active^+^me

Active^+^me is a medically certified (CE marked Class I) telemetry device (ISO 13485:2016) and has a fully customisable suite of lifestyle education (e.g. weight management) and behaviour change support, live exercise classes, physical activity, health monitoring tools, and medication diaries. The platform was designed using the principles of behaviour change described by Abraham & Michie [12]. However, a healthcare professional can decide what resources to provide to patients and when they are provided. This includes which behaviour change techniques to use. Patients were provided with Active^+^me, via post, when they enrolled on to CR. An instruction manual and DVD explaining how to set up the devices were provided. Patients using Active^+^me were also provided with a physical activity tracker, automated blood pressure monitor with heart rate detection, pulse oximeter, and body mass scales. All devices were linked using Bluetooth to a smart device through an application downloaded from the Android (Mountain View, California, USA), Kindle Fire (Seattle, Washington, United States), or Apple (Cupertino, California, USA) app stores. Healthcare professionals communicated with patients throughout the programme, monitored patient progress towards achieving goals, and patient engagement with CR using data transmitted from their accessory devices to a personal computer terminal. Healthcare professionals reviewed patient progress at least once per week and communicated with patients at least once every 3 weeks. Technical support was provided throughout the CR programme by Aseptika Ltd.

### Patient activation

Active^+^me is designed to increase patient activation, a term describing the knowledge, skill, and confidence a patient has to manage their health [7]. Patient activation is measured using the 13-item PAM short-form questionnaire [8], scored on a theoretical scale from 0 to 100. Scores are categorised into one of four patient activation levels, where 1 and 4 denote the lowest and highest level of patient activation, respectively [7, 8]. Level 1 (Scores of 0.0 to 47.0) highlights disengagement and disbelief about the patient’s own role in self-management. Level 2 (scores of 47.1 to 55.1) indicates an increasing awareness, confidence, and knowledge in self-management tasks, however large gaps in their ability to manage their own health remain. Level 3 (scores of 55.2 to 72.4) shows a patient’s readiness and taking action and level 4 (scores of and 72.5 to 100) suggests that patients have adopted new behaviours and maintaining these is a priority [13]. Change in PAM score (0–100) was the primary outcome measure for this study. Patients completed a PAM questionnaire when they enrolled on the Active^+^me programme and at the end of the eight-week CR programme.

### Anthropometric measurements

Face-to-face assessments were suspended due to COVID-19 restrictions [2] so height (cm) and waist circumference was measured by patients, at home, using an inflexible tape measure. Patients were instructed to take their waist circumference measurements at the height of the navel. Resting blood pressure and heart rate measurements were measured using the automated blood pressure machine provided. Patients were asked to sit for at least 5 min before taking their resting blood pressure and heart rate measurements. Body mass was measured using the scales provided. Body mass was divided by height squared, and expressed as body mass index (BMI; kg^.^m^2^).

### Physical activity measurements

Physical activity was measured using the wrist-worn accelerometer which recorded daily minutes of participated physical activity and total daily step count. The activity tracker did not measure exercise intensity so patients also completed a Total Activity Measure 2 (TAM2) questionnaire [14] at the start and end of the CR programme. The TAM2 questionnaire has been validated in patients with heart disease [14] and can be used to estimate how many Metabolic Equivalent of Task-Minutes (MET-minutes) of physical activity a patient completes each week. MET-minutes provide an estimated composite score of physical activity dose characteristics, including intensity, duration, and frequency [14].

### Psychosocial health questionnaires

At the start and end of the CR programme, health care professionals provided each patient with a Patient Health Questionnaire (PHQ) [15], Generalised Anxiety Disorder (GAD) Questionnaire [16], and the Work and Social Adjustment Scale (WAS) [17], to measure changes in depression [15] and anxiety [16] symptoms as well as health-related quality of life [17]. These were dispatched and returned using postal services.

### Adverse events

Adverse events were reported to assess the benefit and risk profile of the intervention. Serious adverse events were defined as any event or reaction that resulted in death, life-threatening illness, hospital admission or prolongation of existing hospitalisation, persistent or significant disability or incapacity [18]. Adverse events were defined as any untoward medical event that occurred during activities required for the study [18], irrespective of whether they were thought to be related to the intervention.

### Interviews

Patient interviews lasted up to 45 min and were conducted after completion of CR by a single member of the research team (KC). A representative sample of patients were recruited using heterogeneous purposive sampling; cardiac diagnosis, age, sex, ethnicity, and follow-up PAM score were considered. Recruitment for interviews stopped when data saturation was met [19]. Data saturation was reached when no new information was attained. A member of the research team contacted patients to explain the purpose of the interviews and obtain additional informed consent. Interviews were conducted using video conferencing software, supported by a topic guide focusing on patient’s experiences of the Active^+^me programme (Table 1). At study completion, healthcare professionals involved with the delivery of Active^+^me were also interviewed for up to 60 min, supported by a topic guide (Table 1).

**Table 1:** Active^+^me evaluation topics for patients and healthcare professionals

Patients	Healthcare professionals
• Recruitment and set up process • Experience of Active^+^me • Active^+^me resources • Usage of Active^+^me • Perceived effectiveness • Self-efficacy and Active^+^me • Health and behaviour change optimism	• Attitude towards Active^+^me for patients • Impact of Active^+^me on clinical practice • Impact of Active^+^me on patients • Unintended consequences

Interviews were analysed using thematic analysis [20]. Audio recordings were transcribed verbatim by an external transcription company for analysis. Transcripts were read, and re-read, to develop familiarity with the data. The transcripts were uploaded to NVivo version 12 (QSR International Pty Ltd., 2019). Sections of raw data that were of interest were highlighted and assigned an initial code. Responses were coded inductively. Once transcripts had been coded the raw data extracts for each theme were re-read, merged, refined, or removed, as appropriate. GF led the interpretation of the data and KC shared reflective notes from each of the interviews to add richness to the themes identified [21]. Concepts were allocated to higher and lower order themes.

### Sample size

Previous data suggests that CR, delivered using telemetry, leads to a 4.8 unit, within group, increase in PAM scores (95% CI: 1.6 to 8) [22]. We converted the 95% CI in to standard deviation (±) using the equation published by the Cochrane Collaboration [23]. The mean change in PAM was 4.8 ± 6.6. These data were used in a sample size calculation, performed using G*Power 3.1 [24]. The significance threshold was set at *P* = 0.05 and the power was set to 99%. The required sample size was 38. Based on previous data from our group [25] we estimated that there would be up to 20% attrition. Thus, the sample size was set at *n* = 46 participants.

### Statistical analysis

Statistical analysis was conducted using SPSS version 24 (IBM, New York, NY, USA). Normality was assessed visually and by using the Shapiro-Wilk test. Categorical data are presented as frequency and percentages. Continuous normally distributed data are presented as mean with 95% confidence intervals. Non-normally distributed continuous data are presented as median with minimum and maximum values. Where missing data or participants were lost to follow-up the last observation was carried forward. A per-protocol analysis was also conducted on the primary outcome measure (PAM). Planned sub-group analysis was conducted on patients at high, moderate, and low risk of exercise-induced cardiovascular events, as defined by established guidelines [5]. To avoid type II error, sub-group analysis was only conducted on the primary outcome measure (PAM score). For parametric data, group differences were assessed using a One-Way ANOVA. A Kruskal-Wallace test was used for non-parametric data. Differences in continuous, normally distributed, paired data were assessed using paired sample t-tests. A Wilcoxon signed-rank test was used to assess differences non-parametric paired data. A Chi-squared test was used to assess differences between categorical variables. Where cells had an expected count < 5, Fishers Exact Test was used. Significance was set at *P* < 0.05. Baseline values were not used as covariates in any analysis. Effect sizes for ANOVA, and Wilcoxon signed rank tests, were calculated as η^2^, where 0.01, 0.06, and 0.140 denoted small, moderate, and large effect sizes, respectively [26]. Effect sizes for t-tests were calculated using Cohen’s D formula [27]. Effect sizes for Small, medium, and large effect sizes for Cohens were 0.2, 0.5, and 0.8, respectively [27].

## Results

Patient characteristics are shown in Table 2. The number of complete responses to each outcome is shown in Appendix 2. There were 154 patients referred for CR. Forty-six (29.9%) patients were given Active^+^me and were included in the evaluation (Age: 60.4: 95% CI 57.1 to 63.6 years: BMI 27.9: 95% CI 26.4 to 29.5 kg^.^m^− 2^; 78.3% male). Reasons for patients not using Active^+^me included lacking the required smart device or internet connection, patients believing they already had tech similar technology such as blood pressure monitors and activity trackers, patients not being interested in using Active^+^me, and language barriers. In some instances, clinicians declined to offer Active^+^me to some patient because they felt it was not suitable for them. The number of patients citing each reason for declining was not documented, nor was the average time to follow-up.

**Table 2:** Patient characteristics (mean; 95% confidence intervals)

Characteristic	All	High Risk	Moderate Risk	Low Risk	*P*-Value	Effect Size
Number of participant (% male)	46 (78.3)	10 (60.0)	13 (76.9)	23 (87.0)	0.224	–
Age (years)	60.4 (57.1 to 63.6)	64.0 (56.4 to 71.6)	62.4 (55.1 to 69.7)	57.7 (53.3 to 62.0)	0.232	0.066
Median Baseline PAM Scores (Range)✝	65.5 (51.0 to 100)	61.9 (51.0 to 91.0)	58.1 (51.0 to 85.0)	65.5 (51.0 to 100)	0.180	0.261
Daily Steps	8312.6 (7314.3 to 9571.8)	5482.3 (3666.6 to 7298.1)^a^	7935.9 (5989.9 to 9882.0)	10,028.0 (8450.0 to 11,611.0)^a^	0.002*	0.286
Daily Physical Activity Duration (Minutes)	94.7 (81.1 to 109.8)	62.4 (42.1 to 82.7)^a^	94.1 (69.2 to 119.0)	111.0 (88.6 to 133.4)^a^	0.020*	0.196
Systolic Blood Pressure (mmHg)	125 (120 to 130)	129 (115 to 143)	115 (103 to 127)	128 (122 to 133)	0.059	0.135
Diastolic Blood Pressure (mmHg)	75 (71 to 79)	73 (62 to 84)	69 (61 to 77)	75 (71 to 79)	0.120	0.103
Resting Heart Rate (bpm) ✝	62 (44 to 93)	62 (44 to 93)	64 (47 to 83)	62 (44 to 93)	0.358	0.066
Body Mass Index (kg^.^m^−2^)	27.9 (26.4 to 29.5)	30.6 (25.9 to 35.4)	25.6 (23.1 to 28.0)	28.0 (26.1 to 29.8)	0.060	0.131
Waist Circumference (cm)	92.8 (82.6 to 102.9)	98.9 (83.1 to 114.7)	99.1 (88.4 to 109.8)	96.3 (84.1104.9)	0.926	0.008
**Ethnicity**						
White British (%)	27 (80.4)	9 (90.0)	10 (76.9)	18 (78.3)	0.534	–
Any other White background (%)	3 (6.5)	0 (0.0)	1 (7.7)	2 (8.7)	0.534	–
Black Caribbean (%)	1 (2.2)	0 (0.0)	0 (0.0)	1 (4.3)		
Indian (%)	1 (2.2)	1(10.0)	0 (0.0)	0 (0.0)		
Any other Asian Background (%)	1 (2.2)	0 (0.0)	1 (7.7)	0 (0.0)		
Not reported (%)	3 (6.5)	0 (0.0)	1 (7.7)	2 (8.7)		
**Primary Reason for Referral**						
Myocardial infarction (%)	25 (54.3)	6 (60.0)	4 (30.8)	15 (65.2)	0.016*	
Elective Percutaneous coronary intervention (%)	10 (21.7)	1 (10.0)	6 (46.2)	3 (13.0)		–
Chronic heart failure (%)	3 (6.5)	3 (30.0)	0 (0.0)	0 (0.0)		
Coronary artery bypass graft (%)	4 (8.7)	0 (0.0)	2 (15.4)	2 (8.7)		
Atherosclerosis (%)	2 (4.3)	0 (0.0)	0 (0.0)	2 (8.7)		
Arrhythmia (%)	1 (2.2)	0 (0.0)	1 (7.7)	0 (0.0)		
Valve surgery (%)	1 (2.2)	0 (0.0)	0 (0.0)	1 (4.3)		

*PAM* Patient Activation Measure

✝ = Non-Parametric Analysis; * significant difference; a = significant difference between high risk and low risk patients

Most patients included for analysis were White British (*n =* 37; 80.4%) and referred following a MI (*n =* 25; 54.3%). Half (*n =* 23; 50.0%) were classified as ‘low risk’. The low risk group had the largest proportion of patients with a PCI. The high risk group had the largest proportion of patients with CHF, or CABG. Three patients were lost to follow-up (6.6%), of which, two (4.4%) discontinued Active^+^me and one (2.2%) was discharged from CR early because the team were unable to make contact with them. Patients in the low risk group completed more daily steps (*P* = 0.002) and accumulated more minutes of daily physical activity at baseline (*P* = 0.020), compared to patients in the high risk group (Table 2). There were no adverse events during the evaluation.

**Fig. 1:**
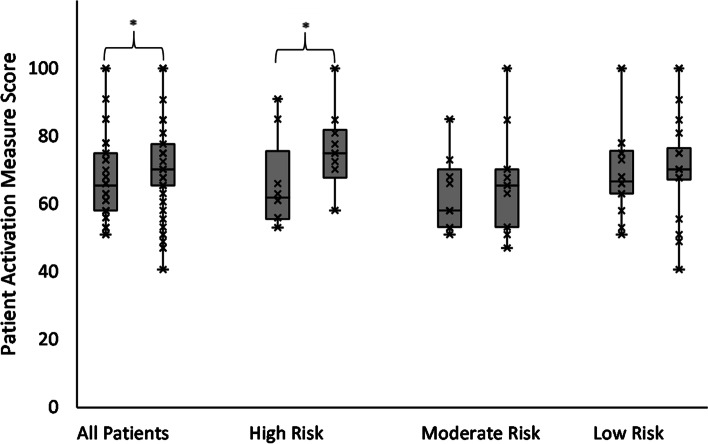
Changes in patient activation measures scores among all patients (left), high risk patients (second from left), moderate risk patients (second from right), and low risk patients (right). *significant difference

**Fig. 2:**
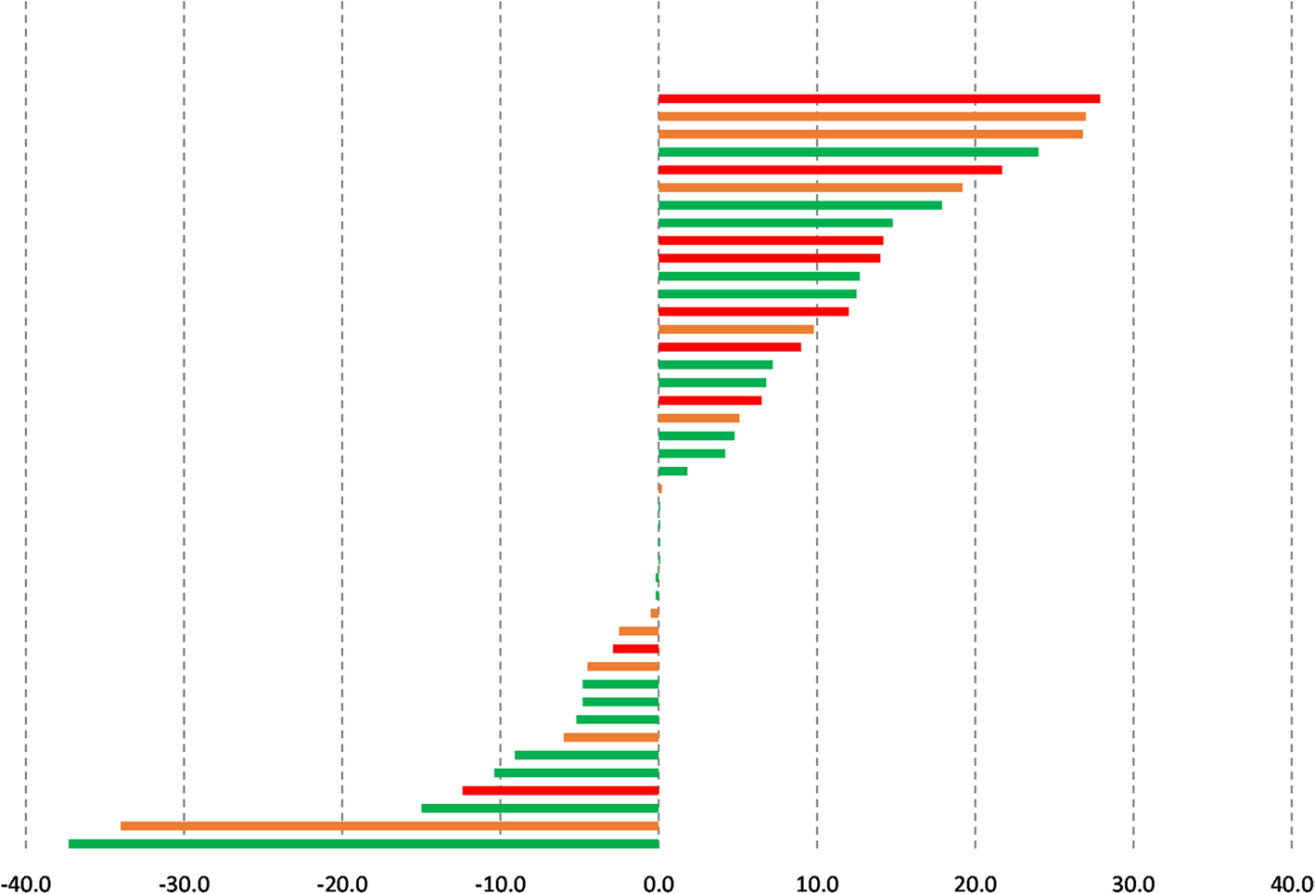
Change in patient activation measure scores in low (green bars), moderate (orange bars), and high risk patients (red bars)

Most patients were categorised as PAM level 3 (50.0%) or 4 (30.4%) at baseline (Table 3). Patient PAM scores (Fig. 1) increased from 65.5 at baseline (range: 51.0 to 100.0) to 70.2 after CR (40.7 to 100.0; *P* = 0.039; η^2^ = 0.101). Carrying the last observed PAM score forward did not change this. Twenty-seven (*n =* 23; 53.4%) patients had higher PAM scores after using Active^+^me. Four (*n =* 4; 9.3%) and 16 (37.2%) patients had no change in PAM scores or lower PAM scores after CR, respectively (Fig. 2). Sub-group analysis showed that PAM scores in high risk patients (*n =* 10) increased from 61.9 (range: 53.0 to 91.0) to 75.0 (range: 58.1 to 100.0; *P* = 0.044; η^2^ = 0.452). The PAM scores of patients in moderate (baseline 58.1; 95% CI 51.0 to 100.0; after CR 65.5; 95% CI 47.0 to 100.0; *P* = 0.441; η^2^ = 0.066) and low risk groups did not change (baseline 66.7; 95% CI 51.0 to 100; after CR 70.2 95% CI 40.7 to 100.0; *P* = 0.522; η^2^ = 0.020; Fig. 1). The proportion of patients reporting an increase in PAM scores was largest in high risk patients (*n =* 7; 70%), followed by moderate (*n =* 6; 54.5%) and low risk patients *(n =* 6; 45.4%; Fig. 2). There were no changes in PAM levels (1 to 4) overall, or within the different risk groups (*P* = 0.107).

**Table 3:** Changes in outcome measures with Active^+^me (mean; 95% Confidence Intervals)

Variable	Baseline	Follow-up	***P***-Value	Effect Size
PAM Scores✝	65.5 (51.0 to 100.0)	70.2 (40.7 to 100.0)	0.039*	0.101
Patients with PAM Level 1 (%)	0 (0.0)	2 (4.3)	0.107	–
Patients with PAM Level 2 (%)	6 (13.0)	4 (8.7)		
Patients with PAM Level 3 (%)	23 (50.0)	20 (43.5)		
Patients with PAM Level 4 (%)	14 (30.4)	17 (37.9)		
Daily Steps	8312 (7314 to 9571)	8484 (7020 to 9814)	0.734	0.045
Daily Physical Activity Duration (Minutes)	94.7 (81.1 to 109.8)	101.7 (82.0 to 119.0)	0.296	0.100
Body Mass Index (kg^.^m^2^)	27.9 (26.4 to 29.5)	27.7 (26.1 to 29.3)	0.126	0.067
Resting Systolic Blood Pressure (mmHg)	125 (120 to 130)	119 (115 to 122)	0.023*	0.445
Resting Diastolic Blood Pressure (mmHg)	75 (71 to 79)	72 (69 to 75)	0.184	0.234
Resting Heart Rate✝ (bpm)	62 (44 to 93)	64 (46 to 87)	0.149	0.053
Waist circumference (cm)	92.8 (82.6 to 102.9)	85.3 (79.1 to 96.2)	0.026*	0.506
PHQ Questionnaire✝	4.0 (0.0 to 23.0)	2.0 (0.0 to 19.0)	0.619	0.016
GAD Questionnaire✝	3.0 (0.0 to 21.0)	1.5 (0.0 to 14.0)	0.693	0.010
WSA Scale Score✝	4.0 (0.0 to 24.0)	1.0 (0.0 to 23.0)	0.906	0.001
TAM2 Score✝ (MET-minutes per week)	1557.5 (245.0 to 5355.0)	3363.2 (105.0 to 12,360.0)	< 0.001*	0.419

*PAM* Patient Activation Measure, *PHQ* Patient Health Questionnaire, General Anxiety Disorder, *WSA* Work and Social Adjustment, *TAM2* Total Activity Measurement, *MET* Metabolic Equivalents

✝ = Non-Parametric Analysis; * significant difference

### Physical activity status and cardiometabolic risk factors

Table 3 shows changes in physical activity and cardiometabolic risk factors. The total duration of physical activity, measured using the physical activity tracker, did not change (mean change: 7.0 min; 95% CI − 6.4 to 20.5; *P* = 0.296; *d* = 0.100). However, self-reported weekly physical activity, estimated using the TAM2 questionnaire, increased by 1422.0 MET-minutes (range: − 2495.0 to 9840.0 (*P* < 0.001; η^2^ = 0.419). More patients reported participating in 150 min of moderate intensity physical activity per week at follow-up (*n =* 30; 69.8%) than baseline (*n =* 14; 31.0%), but this was not significant (*P* = 0.147). Two patients participated in more than 75 min of vigorous physical activity at baseline (4.3%), and follow up (4.7%). Systolic blood pressure was lower at follow-up, compared to baseline (*P* = 0.023; *d* = 445), but diastolic blood pressure did not change (*P* = 0.184; *d* = 0.053). Waist circumference measurements were also smaller at follow-up, compared to baseline (*P* = 0.026; *d* = 0.506) but BMI did not change (*P* = 0.126). However, there was a moderate effect size for lower body mass (η^2^ = 0.067). Carrying the last observation forward did not change the significance of the results. However, the change in waist circumference was smaller to − 2.5 cm (95% CI − 0.2 to − 4.8 cm; *P* = 0.033; *d = 0.*163).

### Psychosocial questionnaires

The number of patients returning baseline and follow-up psychosocial questionnaires was small (*n* = 17; Appendix 2). There were no changes in any psychosocial measurements, measured using questionnaires (all *P* > 0.05; Table 3).

### Qualitative results

Nine males and four females were interviewed (total *n =* 13; median age: 62.0 years; range 46 to 79 years). Eleven (84.6%) were White British, one was Asian (7.7%), and one Black Caribbean (7.7%). Interviews were conducted with three CR Exercise Professionals. Three higher-order themes were identified (i) Facilitators of Active^+^me participation and adherence (ii) Barriers of Active^+^me participation and adherence (iii) Level of engagement with Active^+^me components. Further explanation of the themes, and additional quotes, are shown in Appendix 3.

#### Theme 1: Facilitators of Active^+^me participation and adherence

##### Subtheme 1.1: Perceived usefulness of Active^+^me

Patients and professionals believed Active^+^me participation was important during the COVID-19 pandemic because face-to-face CR was suspended. The healthcare professionals recognised the importance of the programme, and considered this an opportunity to build good communication with patients during this time, whilst offering confidence that patients are ‘safe’ whilst exercising independently at home.

*“…It allowed us to get a lot more information on patients that we might not have got during COVID-19 period”.* (Healthcare professional 3)

##### Subtheme 1.2: Programme benefits

All patients discussed at least one benefit of participating and described an improvement in health-related confidence, physical activity levels, ability to manage their health, and/or psychological wellbeing. Ten patients (76.9%) said their ability to manage their health had improved since participating in Active^+^me. Patients who discussed more benefits of attending the programme had larger increases in PAM scores.

*“I am a lot more aware of my health issues… I became a lot more involved in trying to change that for the better, rather than for the worse”.* (Female, 47 years)

##### Subtheme 1.3: Self-motivation

Goal setting, self-monitoring and belief in capability (self-efficacy) were all discussed as factors which influenced participation. Patients considered a level of self-motivation was required in order to ‘get the most out of the programme’, predominantly because the programme was carried out independently in their own home.

*“…II do my 10,000 steps and I haven’t missed a day, since I got it, every single day at the moment I’ve done the 10,000* ^*+*^ *steps”.* (Male, 71 years)

#### Theme 2: Barriers to Active^+^me participation and adherence

##### Subtheme 2.1: Perceived health status

Nine out of the 13 patients (69.2%) who engaged with the Active^+^me programme discussed other chronic health conditions which impacted some patients’ ability, and perceived capability, to participate with some components of the programme. These included musculoskeletal conditions, strokes, anxiety, and depression. Those with co-morbidities were a mixture of low, moderate and ‘high risk’ patients. Not all patients who had co-morbidities were categorised as ‘high risk’. Patients who had a co-morbidity were more likely to voice their apprehension or inability to engage with certain aspects of the programme and considered themselves less capable in the exercise component.

*“…I had other things that were other issues, particularly this leg problem…which nobody can solve, it ruined my experience to a certain extent. It stopped me being able to get the full use out of it”.* (Male 79 years)

##### Theme 2.2: Increased burden

Even though Active^+^me was designed to provide flexibility in the scheduling of each of the programme components, including exercise and physical activity, some patients had family or work commitments and perceived this as a barrier to participation.

*“Presently I am caring for my husband who has slight dementia and with the Type 1 diabetes that I have, life gets a bit tricky”.* (Female, 67 years)

Health care professionals considered time and other work commitments as a barrier to engaging with Active^+^me. There was a perception that Active^+^me needed to be better integrated into the processes of usual CR. However, over the project duration, the processes and procedures were streamlined to create better ways of working.

*“…it was at times quite time consuming for clinicians…it did take up quite a bit of time with reviewing data in certain patients that needed more support. As the months went by, we learnt the best way of doing things and processes evolved and got better and better to make it work for both us and the patients”.* (Healthcare professional 2)

#### Theme 3: Level of engagement with active^+^me programme components

##### Subtheme 3.1: Self-monitoring components and healthcare professional support

All 13 patients engaged with at least one element of Active^+^me. The most utilised components were the physical activity tracker, the blood pressure monitor and scales. Ten (76.9%) of 13 patients engaged with the physical activity or step tracking component, however, three (23.1%) patients lacked confidence in their ability to complete the exercise components believing that exercise would have a negative effect on their health. Proportionately, high and medium risk patients were more likely to discuss feeling disengaged when they used the smart device application due to the perceived level of ‘competition’ with other patients.

*“It made me feel bad… I couldn't relate to people walking 10,000 steps in a day”.* (Male, 79 years)

A ‘one size fits all approach’ was not considered appropriate. Instead, a personalised approach was recommended by health care professionals and patients. Providing self-monitoring equipment and contact time support according to patient need.

*“I’d be maybe personalising a little bit more to each patient… so definitely look at that side of things as to who gets what and what they need to be reviewing”.* (Heath care professional 2)

All interview patients discussed the importance of having a healthcare professional who was aligned with the programme to advise on health statistics, technical support and safety. Health care professional experience, knowledge of condition and reassurance were also viewed as especially important to patients. Healthcare professional support was considered valuable for monitoring patients, offering reassurance and assisted with continuity of care.

*“It was very useful and enriching for my role to be able to speak to them and I could hear the nerves in their voice sometimes and just to say, you know, you’re not alone through the process*”. (Health Care Professional 1)

##### Subtheme 3.2: Education

Seven (53.8%) patients discussed some engagement with the lifestyle education component of Active^+^me. Patients who used the information found it helpful, although potentially overwhelming in quantity.

*“…when you go through an experience like I did you are bombarded with information from many different angles, and a lot of it obviously is repeated, which is good because it means it gets in there, but you are, bombarded”.* (Male, 54)

Healthcare professionals said further development of the lifestyle education component would enhance this aspect of the programme, and help patients self-manage their condition, in the longer-term.

*“I think from the education point of view… if we were to fully engage and develop it, it’s a really good resource that patients can have available for them to have whenever they need it to look through in the long term”.* (Health Care Professional 2)

## Discussion

In 2020, restrictions on face-to-face contact, due to COVID-19, led to a rapid increase in the use of technology to deliver CR [3]. Some technologies, including Active^+^me, had not been evaluated in patients with heart disease. We are the first to evaluate Active^+^me in a cohort of patients participating in a Phase III CR programme during the COVID-19 pandemic. Interviews showed that patients engaged with Active^+^me and that they were more confident at managing their own health after completing the programme. This was reflected by an increased in PAM score, our primary outcome measure, after participating in CR with Active^+^me. Interestingly, PAM increased most in patients at high risk of exercise-induced cardiovascular events. Changes in PAM scores were accompanied by reductions in systolic blood pressure, waist circumference, and increased self-reported physical activity. However, several patients reported lower PAM scores after the intervention which may indicate a need to refine the content provided to some patients. We were unable to draw any conclusions on whether participation in CR with Active^+^me improved psychosocial health due to the low number of questionnaire responses (Appendix 2).

### Patient activation

One aim of CR is to help patients learn how to self-manage their heart condition [6]. We found that PAM scores, a metric that quantifies the skill, confidence and knowledge a patient has to manage their health [7], increased after CR with Active^+^me (from 65.5 to 70.2). This is consistent with previous data showing that PAM scores increased by 4.2 points after hospital-based CR, and 4.8 points after telemetry-based CR [22]. The overall change in PAM score in our study appeared to be driven by increases among high risk patients (61.9 to 75.0). No adverse events were reported. These findings provide reassurances about the safety and benefit of remotely delivered CR for patients, including high risk patients, and should help reverse the trend of digital exclusion for this group of patients [3].

The reason for greater improvements in PAM scores among high risk patients, but not low and moderate risk patients, is unclear. Lower baseline PAM scores in high risk patients might have suggested greater potential to increase PAM scores. However, baseline PAM scores were similar in all risk groups (Table 2). Thus, changes in PAM score appear to be independent of baseline PAM scores, between risk groups, in this instance.

In qualitative interviews, patients with larger increases in PAM scores generally discussed more meaningful health benefits following CR with Active^+^me. Therefore Active^+^me, seems to be more beneficial for high risk patients. These patients may be more motivated to learn about how to manage their health, compared to moderate and low risk individuals. This may be because they are more aware of their poor health and are more motivated to see an improvement in their health. However, this is speculative.

### Cardiovascular risk factors

Blood pressure in our cohort of patients was well managed from the outset. However, cardiovascular risk associated with hypertension exists on a continuum [28]. The risk of cardiovascular events is halved with each 20 mmHg reduction in systolic blood pressure, down to ~ 115 mmHg [28]. The reduction in systolic blood pressure, from 125 mmHg to 119 mmHg, is consistent with changes following comprehensive CR (− 3.2 mmHg; 95% CI − 5.6 to − 0.8) [29]. Importantly, the − 3.2 mmHg reduction in systolic blood pressure may *contribute* towards the 37% reduction in all-cause mortality observed in patients participating in comprehensive CR [29]. The systolic blood pressure reduction of ~ 6 mmHg following participation in CR with Active^+^me may therefore be meaningful.

The reasons for the reduction in systolic blood pressure are likely to be multifactorial. For example, Active^+^me provides support to encourage medication adherence. Therefore, better adherence to anti-hypertensive medication may have contributed to a reduction in systolic blood pressure. However, this was not measured in this study. Patients using Active^+^me also reported a reduction in waist circumference, a surrogate of abdominal obesity. Weight loss > 5% is associated with a reduction in systolic blood pressure [30]. However, unlike waist circumference, BMI was unchanged after Active^+^me. This discrepancy may be due to patients measuring their own waist circumference. Waist measurements may therefore be inaccurate. It may also be due to incomplete data for waist circumference measurements (Appendix 2). Notably, carrying the last observation forward resulted in a smaller reduction in waist circumference measurements than with the raw analysis. An alternative explanation for the reduction in blood pressure could be the increase self-reported physical activity levels. Increased participation in physical activity levels is associated with a reduction in systolic blood pressure [31]. However, changes in self-reported physical activity were not reflected in accelerometer-derived measurements of physical activity. This may be because the physical activity tracker did not measure exercise intensity and/or because it only detected steps. Activities such as cycling may not have been recorded. Self-reported physical activity may have increased because this measure captured activities that were not recorded by the activity tracker and/or because it captured changes in intensity of physical activity. Interviews also suggested that some patients did not use the physical activity tracker which could have resulted in missing data.

### Active^+^me adherence

In 2019, the UK’s National Audit for Cardiac Rehabilitation (NACR) reported that 50% of eligible patients chose to participate in CR. Of these, 77% completed their CR programme [32]. Whilst only ~ 30% of patients referred for CR used Active^+^me, completion of CR with Active^+^me was 93.4%. These data suggest that Active^+^me could help increase completion rates of CR and therefore maximise the benefit of CR to the patient. The high programme completion rates also indicate that patients are more likely to complete a CR programme if they chose the mode of delivery. However, qualitative interviews showed that some patients disengaged with certain elements of Active^+^me. More research and intervention development is needed to improve patient fidelity to the different components of Active^+^me.

### Limitations and conclusion

Active^+^me with routine CR is acceptable to patients and healthcare professionals. Overall, findings support that patients have better skills, knowledge, and confidence to manage their heart condition after completing CR with Active^+^me. Patients at high risk of cardiovascular events seemed to benefit the most. Improvements in patient activation were associated with lower systolic blood pressure, and increased self-reported physical activity levels, in the short-term. Thus, current evidence supports the use of Active^+^me for patients in CR. However, as this was a cohort evaluation of newly adopted standard practice, some secondary outcome measures collected as part of routine care were incomplete. These included waist circumference measurements and psychosocial questionnaires (Appendix 2). The low number of responses could explain why no improvements in psychosocial measurements were reported. Further, the number of participants in sub-groups was small and unevenly distributed. Conclusions regarding these outcome measures should therefore be interpreted with caution. Additionally, we did not collect control data, and data were only collected from one site. The generalisability of our findings are therefore unclear. Finally, although we obtained qualitative data about whether patients engaged with Active^+^me, we did not collect quantitative data on app usage or treatment fidelity. Further large-scale controlled studies are needed to confirm the benefit of using Active^+^me to support remotely delivered CR.

## Supplementary Information

**Additional file 1 **: **Appendix 1.** STROBE Statement—checklist of items.

**Additional file 2 **: **Appendix 2.** Number of responses for each outcome measure.

**Additional file 3.** Supplementary explanation of themes and additional quotes.

## Data Availability

Reasonable request for the original anonymised data set can be made by e-mailed the corresponding author directly, or by e-mailing the Sheffield Hallam University Research Data Archive (SHURDA) administrator at shurda@shu.ac.uk.

## Abbreviations

BMI: Body Mass Index
CABG: Coronary Artery Bypass Graft
CHD: Coronary Heart Disease
CHF: Chronic Heart Failure
CI: Confidence Interval
CR: Cardiac Rehabilitation
HR: Hazard Ratio
MET-minutes: Metabolic Equivalent of Task-Minutes
MI: Myocardial Infarction
PAM: Patient Activation Measure
PCI: Percutaneous Coronary Intervention
OR: Odds Ratio
TAM2: Total Activity Measure 2
