# Supplementary material for: Changes in patient activation following cardiac rehabilitation using the Active+me digital healthcare platform during the COVID-19 pandemic: a cohort evaluation
Source: BMC Health Serv Res. 2021 Dec 24;21:1363. doi: 10.1186/s12913-021-07363-7 (PMC8703006; doi:10.1186/s12913-021-07363-7)
Supplement: Supplementary file 2 — Additional file 2 : Appendix 2. Number of responses for each outcome measure. [file 12913_2021_7363_MOESM2_ESM.docx]

**Appendix 2 – Number of responses for each outcome measure**

| **Outcome Measure** | **Baseline Responses** | **Follow-up Responses** | **Complete Responses** |
| --- | --- | --- | --- |
| PAM Score | 43 | 43 | 43 |
| PAM Level | 43 | 43 | 43 |
| Daily Steps | 39 | 38 | 37 |
| Daily Physical Activity Duration (minutes) | 39 | 38 | 37 |
| Systolic Blood Pressure (mmHg) | 42 | 44 | 42 |
| Diastolic Blood Pressure (mmHg) | 42 | 44 | 42 |
| Resting Heart Rate (bpm) | 38 | 41 | 38 |
| Body Mass Index (kg^.^m^2^) | 43 | 43 | 43 |
| Waist Circumference (cm) | 21 | 19 | 10 |
| PHQ Questionnaire | 29 | 27 | 17 |
| GAD Questionnaire | 29 | 27 | 17 |
| WSA Scale Score | 29 | 26 | 17 |
| TAM2 Score | 41 | 41 | 41 |
