# Supplementary material for: Changes in patient activation following cardiac rehabilitation using the Active+me digital healthcare platform during the COVID-19 pandemic: a cohort evaluation
Source: BMC Health Serv Res. 2021 Dec 24;21:1363. doi: 10.1186/s12913-021-07363-7 (PMC8703006; doi:10.1186/s12913-021-07363-7)
Supplement: Supplementary file 3 — Additional file 3. Supplementary explanation of themes and additional quotes. [file 12913_2021_7363_MOESM3_ESM.docx]

**Supplementary explanation of themes and additional quotes**

| **Higher-order themes** | **Lower-order themes** | **Brief description** | **Quote examples** |
| --- | --- | --- | --- |
| 1. **Facilitators to Active^+^me participation and adherence** | *1.1: Perceived usefulness of Active^+^me*  *Patients and professionals’ perceived level of programme usefulness.* | Patients and professionals viewed Active^+^me as useful for assisting with continuity of care particularly during COVID-19.  Patients expressed almost entirely positive attitudes towards the perception that Active^+^me was important/useful as part of their treatment. | *“When patients weren’t having as much follow up with maybe their GP, or cardiologist because of COVID-19…at least we were able to give them little bit of support…we knew that they were getting those values checked properly”.*  (Health Care Professional 2)  *“…It allowed us to get a lot more information on patients that we might not have got during coronavirus period”.*  (Health Care Professional 3)  *“Given the circumstances, the timing of it (Active^+^me) keeps the show on the road”.* (Male, 55 years*)*  *“…during this pandemic of course it's very difficult for everybody but I used to get a phone call every three weeks and run through the whole programme”.*  (Male, 71 years)  *“I considered it an essential part of treatment”.*  (Male, 79 years)  *“I was looking for some sort of support…something to make me at ease with what I was trying to do. Because if you’re left to your own devices, nothing much happens, and you don’t do anything”.*  (Male, 71 years) |
|  | *1.2: Programme benefits*  *Factors which patients and professionals deemed as beneficial when taking part with the programme.* | Patients and professionals wanted to see the benefits of engaging with Active^+^me and to know that it was going to be of value. All patients discussed at least one participatory benefit. | *“Walking was an issue for me several months ago, I couldn't walk any great distance without either getting pain in my hip or short of breath… now I walk more, and I don't get breathless”.*  (Male 71 years)  *“…it made me feel a lot more confident that the advice I was giving them was appropriate because I knew that this person’s blood pressure was within range and I knew that their weight was coming in the right direction rather than someone just telling me over the phone…we could actually back that up with the data so it gave me confidence that the patients were safe”.*  (Health Care Professional 2)  *“I am a lot more aware of my health issues….my health is usually very poor and I wasn't doing anything really to change that, but I became a lot more involved in trying to change that for the better, rather than for the worse”.*  (9018)  *“It changed my life, you know. I stopped drinking fizzy drinks…I eat a lot more salads, …and I’m cooking myself healthier meals”.*  (9048) |
|  | *1.3: Self-motivation*  *Intrinsic factors that influenced patient motivation to engage with Active^+^me.* | Goal setting, regular self-monitoring, belief in capability (self-efficacy) were all factors that influenced patient motivation. | *“We go out early in the morning and then go out later in the evening and make sure I do my steps. And I haven’t missed a day, since I got it, every single day at the moment I’ve done the steps”.*  (Male, 71 years)  *“…things have become a habit so, yes, I am doing the exercise and moving about and I go for a walk subject to the weather, etc, when I can. So, if I take an hour’s lunch then half of it will be walking just briskly around the area where I work to have a break and to do steps… so yes, it has become a habit”.*  (Male, 73)  “…it depends on your state of mind as well, if you’re not looking forward to it you probably find it more negative than it is but if you’re a fairly positive person, I like to think I am, then you overcome these things and you get on with it as best you can”.  (Male, 73) |
| 1. **Barriers to Active^+^me participation and adherence** | *2.1: Perceived health status*  *A patient's relative level of wellness and illness perception.* | The majority of patients interviewed has discussed other chronic health conditions. Some which impacted their ability and perceived capability to participate with some components of the programme. | *“It's just in my particular instance where I had other things that were other issues, particularly this leg problem, which nobody can solve, it ruined it to a certain extent. It stopped me being able to get the full use out of it. I have to say I felt…a fraud that I was using it and I felt that other people could be making more use of it”.*  (Male, 79 years)  “*I actually did the first part of the exercise one until I then hit a spot where I couldn't do it any further”.*  (Male, 71 years) |
|  | *2.2: Increased burden*  *Patient and health care professional perspectives on Active^+^me impacting their daily responsibilities.* | Some patients and health care professionals discussed how the Active^+^me programme impacted on their daily responsibilities, such as caring and work commitments for patients and other work demands for health care professionals. | *“Presently I am caring for my husband who has slight dementia and with the Type 1 diabetes that I have life gets a bit tricky”.*  (Female, 67 years)  *“We can’t just stop the way that we run to take on this project, it has to be integrated well, and we have to understand the time constraints involved from our end as well”.* (Health Care Professional 3)  *“…because I’m back to work now because I’m doing 10 hours a day, so I can’t do it anymore. I mentioned it to the cardiac rehab team that recently I haven’t done the exercises”.*  (Female, 46 years)  *“Well I find it sometimes took more time than I would have liked. I’ve got other activities as well”.*  (Male, 79) |
| **3: Level of engagement with Active^+^me programme components** | *3.1: Self-monitoring and health care professional support*  *Level of engagement with the self-monitoring equipment including scales, blood pressure monitor, physical activity tracker, app and oxygen saturation* | All patients engaged with a range of the programme components. Both patients and professionals discussed how providing self-monitoring equipment according to each patient need would be more suitable.  Patients valued the communication with health care professionals for medical and technical support as well as reassurance. | *“It’s good to let you know how I’m getting on in my recovery…like how my weight, blood pressure and heartbeat is…it’s good to have it here all linked to the computer”.* (Male, 49 years)  *“I liked the fact that I didn't have to leave home to go anywhere to be sort of checked-out and monitored”.*  (Male, 59)  *“I have found it very useful to enable me to check on blood pressure etc and I would certainly recommend to other patients who have had heart problems like mine. I was pleased to be asked to join this group and found all very useful”.*  (Female, 67 years)  *“…the calls were fantastic… when you felt quite low and down…it was really good to have a call to say, in two weeks they ring and check how you’re doing”.*  (Male, 59 years)  *“It was very useful and kind of enriching for my role to be able to speak to them and I could hear the nerves in their voice sometimes and just to say, you know, you’re not alone through the process*”.  (Health Care Professional 1)  *“I’d be maybe personalising a little bit more to each patient… so definitely look at that side of things as to who gets what and what they need to be reviewing”.*  (Health Care Professional 2) |
|  | *3.2: Education*  Level of engagement with the lifestyle education components of Active^+^me. | The lifestyle education components appeared underutilised and health care professionals believed this area needed more development. | *“I think from the education point of view… if we were to sort of fully engage with that side and look into what’s being…sent out on the app it’s a really good resource that they can have available for them in the long term, to have whenever they need it to look through”.*  (Health Care Professional 2)  *“…when you go through an experience like I did you are bombarded with information from many different angles, and a lot of it obviously is repeated, which is good because it means it gets in there, but you are, you are bombarded”.*  (Male, 54)  *“ …there was a lot of information to take in and it was very useful”.*  (Female, 67 years) |
